# Supplementary material for: Correlation Analysis of Soil Microbial Communities and Physicochemical Properties with Growth Characteristics of Sageretia thea Across Different Habitats
Source: Plants (Basel). 2024 Nov 26;13(23):3310. doi: 10.3390/plants13233310 (PMC11644716; doi:10.3390/plants13233310)
Supplement: Supplementary file 1 [file plants-13-03310-s001.zip › plants-3279976-supplementary.pdf]

SUPPLEMENTARY DATA

Table S1. Duncan's correlation coefficient between soil physicochemical properties and microbial communities (Phyllum) of *S. thea* habitats

	Correlation coefficient (r) [a]						
	Proteo bacteria	Bacter oidetes	Verru comicrobia	Acido bacteria	Actino bacteria	Chloroflexi	Firmicutes
OM	0.050	-0.154	-0.006	-0.341*	0.581**	0.030	-0.344
	(0.396)	(0.216)	(0.488)	(0.032)	(0.000)	(0.439)	(0.183)
Total	0.066	-0.118	-0.070	-0.342*	0.573**	0.073	-0.322
N	(0.365)	(0.274)	(0.356)	(0.032)	(0.000)	(0.353)	(0.199)
Avai.	0.166	0.437*	-0.361*	-0.247	0.265	0.210	-0.272
P	(0.190)	(0.010)	(0.025)	(0.094)	(0.078)	(0.137)	(0.240)
K	0.266	0.267	-0.121	-0.553**	0.456**	0.169	-0.400
	(0.077)	(0.085)	(0.261)	(0.001)	(0.006)	(0.190)	(0.143)
Ca	0.081	0.315	-0.231	-0.573**	0.643**	-0.140	-0.389
	(0.336)	(0.051)	(0.109)	(0.000)	(0.000)	(0.235)	(0.151)
Mg	-0.046	-0.120	0.021	-0.364*	0.490**	-0.027	-0.302
	(0.404)	(0.271)	(0.456)	(0.024)	(0.003)	(0.444)	(0.215)
Na	-0.174	-0.177	-0.038	-0.251	0.439**	0.051	-0.153
	(0.180)	(0.184)	(0.422)	(0.090)	(0.008)	(0.397)	(0.347)
pH	0.058	-0.027	0.096	-0.374*	0.605**	-0.022	-0.364
	(0.380)	(0.447)	(0.306)	(0.021)	(0.000)	(0.454)	(0.168)
CEC	-0.329*	0.170	-0.320*	0.037	0.041	0.026	0.275
	(0.038)	(0.193)	(0.043)	(0.423)	(0.415)	(0.447)	(0.237)
EC	0.388*	0.127	-0.086	-0.663**	0.506**	-0.042	-0.368
	(0.017)	(0.259)	(0.325)	(0.000)	(0.002)	(0.413)	(0.165)

[a] Correlation coefficient (r) written is significantly correlated between the variables compared. Positive values denote positive correlation and negative values denote negative correlation. Values in bracket means p value (** $p < 0.01$, * $p < 0.05$)

Table S2. Duncan's correlation coefficient between soil physicochemical properties and microbial communities (Classes) of *S. thea* habitats

	Correlation coefficient (r) [a]											
	Alphaproteobacteria	Actinobacteria_c	Vicinamibacter_c	Acidobacteriia	Spartobacteria	Betaproteobacteria	Solibacteres	Gammaproteobacteria	Rubrobacteria	Deltaproteobacteria	Thermoleophilia	Blastocatellia
OM	0.238	0.369*	-0.161	0.267	0.052	-0.386*	-0.002	0.152	0.344	-0.338	0.610**	-0.397
	(0.205)	(0.045)	(0.422)	(0.256)	(0.794)	(0.035)	(0.992)	(0.424)	(0.068)	(0.073)	(0.001)	(0.061)
Total N	0.195	0.357	-0.125	0.253	-0.021	-0.332	-0.029	0.206	0.347	-0.353	0.605**	-0.400
	(0.302)	(0.053)	(0.534)	(0.281)	(0.917)	(0.073)	(0.881)	(0.274)	(0.065)	(0.061)	(0.001)	(0.057)
Avai. P	-0.100	0.205	0.087	0.193	-0.311	-0.067	-0.200	0.517**	0.051	-0.115	0.303	-0.031
	(0.600)	(0.276)	(0.666)	(0.414)	(0.108)	(0.724)	(0.298)	(0.003)	(0.791)	(0.554)	(0.110)	(0.890)
K	-0.068	0.322	0.004	-0.260	-0.112	0.100	-0.396*	0.447*	0.292	0.153	0.350	-0.362
	(0.720)	(0.082)	(0.984)	(0.267)	(0.569)	(0.599)	(0.033)	(0.013)	(0.124)	(0.427)	(0.063)	(0.090)
Ca	-0.137	0.398*	0.192	-0.574**	-0.218	0.008	-0.626**	0.234	0.503**	0.202	0.562**	-0.230
	(0.472)	(0.029)	(0.296)	(0.008)	(0.265)	(0.966)	(0.001)	(0.213)	(0.005)	(0.293)	(0.001)	(0.292)
Mg	-0.023	0.198	-0.003	-0.389	0.020	-0.191	-0.249	0.098	0.428*	-0.059	0.487**	-0.265
	(0.905)	(0.293)	(0.990)	(0.090)	(0.919)	(0.311)	(0.193)	(0.607)	(0.020)	(0.760)	(0.007)	(0.222)
Na	-0.090	0.159	0.082	-0.240	-0.042	-0.243	-0.226	-0.021	0.388*	0.025	0.476**	-0.176
	(0.635)	(0.401)	(0.686)	(0.308)	(0.830)	(0.195)	(0.239)	(0.913)	(0.037)	(0.899)	(0.009)	(0.423)
pH	0.296	0.378*	-0.083	0.114	0.131	-0.408*	-0.069	0.079	0.447*	-0.251	0.562**	-0.455*
	(0.112)	(0.040)	(0.682)	(0.633)	(0.506)	(0.025)	(0.722)	(0.679)	(0.015)	(0.189)	(0.001)	(0.029)
CEC	-0.622**	-0.130	0.631**	-0.619**	-0.350	0.195	-0.643**	-0.152	0.152	0.676**	0.036	0.540**
	(0.000)	(0.492)	(0.000)	(0.004)	(0.068)	(0.303)	(0.000)	(0.422)	(0.431)	(0.000)	(0.853)	(0.008)
EC	0.057	0.430*	-0.124	-0.363	-0.068	0.117	-0.400*	0.507**	0.300	0.078	0.358	-0.290
	(0.767)	(0.018)	(0.538)	(0.116)	(0.732)	(0.539)	(0.031)	(0.004)	(0.114)	(0.689)	(0.056)	(0.180)

[a] Correlation coefficient (r) written is significantly correlated between the variables compared. Positive values denote positive correlation and negative values denote negative correlation. Values in bracket means p value ($^{**}\ p < 0.01$, $^{*}\ p < 0.05$)

Table S3. Duncan's correlation coefficient between growth characteristics of *S. thea* and microbial communities (Phyllum) in habitats

	Correlation coefficient (r) [a]						
	Proteo bacteria	Bacter oidetes	Verru comicrobia	Acido bacteria	Actino bacteria	Chloroflexi	Firmicutes
Leaf length	0.294	-0.373*	-0.012	-0.293	0.161	-0.088	0.100
	(0.058)	(0.025)	(0.475)	(0.058)	(0.198)	(0.324)	(0.399)
Leaf width	0.344*	-0.319*	0.078	-0.199	0.083	-0.082	-0.147
	(0.031)	(0.049)	(0.340)	(0.146)	(0.332)	(0.337)	(0.353)
Fruit length	-0.033	-0.290	0.276	-0.048	0.015	-0.359*	0.153
	(0.430)	(0.067)	(0.070)	(0.400)	(0.468)	(0.028)	(0.347)
Fruit width	0.179	-0.179	0.382*	-0.174	-0.109	-0.470**	0.168
	(0.172)	(0.181)	(0.019)	(0.179)	(0.283)	(0.005)	(0.333)
Fruit weight	0.321*	-0.133	0.174	-0.065	-0.265	-0.476**	0.286
	(0.042)	(0.250)	(0.178)	(0.366)	(0.078)	(0.004)	(0.228)
sweetness	0.122	-0.072	-0.068	-0.001	0.361*	0.071	-0.444
	(0.261)	(0.358)	(0.360)	(0.498)	(0.025)	(0.357)	(0.116)
hardness	-0.210	-0.012	-0.051	0.094	0.148	0.330*	-0.438
	(0.133)	(0.476)	(0.394)	(0.311)	(0.218)	(0.040)	(0.119)

[a] Correlation coefficient (r) written is significantly correlated between the variables compared. Positive values denote positive correlation and negative values denote negative correlation. Values in bracket means p value (** $p < 0.01$, * $p < 0.05$)

Table S4. Duncan's correlation coefficient between growth characteristics of *S. thea* and microbial communities (Classes) in habitats

	Correlation coefficient (r) [a]											
	Alphaproteobacteria	Actinobacteria_c	Vicinamibacter_c	Acidobacteriia	Spartobacteria	Betaproteobacteria	Solibacteres	Gammaproteobacteria	Rubrobacteria	Deltaproteobacteria	Thermoleophilia	Blastocatellia
Leaf	0.313	0.356	-0.352	0.447*	-0.002	-0.022	0.196	0.159	-0.039	-0.148	0.092	-0.357
length	(0.092)	(0.053)	(0.072)	(0.048)	(0.993)	(0.909)	(0.307)	(0.402)	(0.842)	(0.443)	(0.635)	(0.095)
Leaf	0.341	0.303	-0.362	0.373	0.090	0.112	0.271	0.127	-0.085	-0.144	-0.011	-0.398
width	(0.065)	(0.104)	(0.064)	(0.105)	(0.647)	(0.555)	(0.154)	(0.502)	(0.663)	(0.455)	(0.957)	(0.060)
Fruit	0.026	0.068	-0.206	-0.358	0.274	0.064	-0.076	-0.219	-0.074	0.174	-0.044	0.278
length	(0.891)	(0.721)	(0.304)	(0.122)	(0.158)	(0.737)	(0.695)	(0.245)	(0.701)	(0.366)	(0.821)	(0.198)
Fruit	0.163	0.035	-0.346	-0.331	0.370	0.119	-0.020	-0.054	-0.156	0.186	-0.231	0.049
width	(0.391)	(0.853)	(0.077)	(0.058)	(0.052)	(0.530)	(0.916)	(0.776)	(0.420)	(0.334)	(0.228)	(0.825)
Fruit	0.135	0.062	-0.289	-0.289	0.155	0.250	0.047	0.102	-0.377*	0.199	-0.345	0.231
weight	(0.478)	(0.746)	(0.144)	(0.217)	(0.430)	(0.183)	(0.811)	(0.593)	(0.044)	(0.301)	(0.067)	(0.290)
sweetness	0.205	0.294	0.278	0.366	-0.050	-0.063	-0.017	-0.011	0.130	-0.056	0.380*	-0.005
	(0.278)	(0.115)	(0.160)	(0.113)	(0.801)	(0.739)	(0.931)	(0.954)	(0.500)	(0.775)	(0.042)	(0.980)
hardness	-0.197	0.014	0.114	0.001	-0.042	0.011	-0.085	-0.171	0.174	0.106	0.072	0.079
	(0.297)	(0.804)	(0.570)	(0.997)	(0.832)	(0.953)	(0.663)	(0.365)	(0.367)	(0.582)	(0.710)	(0.722)

[a] Correlation coefficient (r) written is significantly correlated between the variables compared. Positive values denote positive correlation and negative values denote negative correlation. Values in bracket means p value (** $p < 0.01$, * $p < 0.05$)
